# Protocol for high-throughput DNA methylation profiling in rat tissues using automated reduced representation bisulfite sequencing

**DOI:** 10.1016/j.xpro.2024.103007

**Published:** 2024-04-30

**Authors:** Venugopalan D. Nair, Hanna Pincas, Mary Anne S. Amper, Yongchao Ge, Mital Vasoya, Archana Natarajan Raja, Martin J. Walsh, Stuart C. Sealfon

**Affiliations:** 1 Department of Neurology, Icahn School of Medicine at Mount Sinai, New York, NY 10029, USA; 2 Department of Medicine, Stanford School of Medicine, Stanford, CA 94305, USA; 3 Department of Pharmacological Sciences, Icahn School of Medicine at Mount Sinai, New York, NY 10029, USA

**Keywords:** Genomics, High-Throughput Screening, Molecular Biology

## Abstract

Although reduced representation bisulfite sequencing (RRBS) measures DNA methylation (DNAme) across CpG-rich genomic regions with high sensitivity, the assay can be time-consuming and prone to batch effects. Here, we present a high-throughput, automated RRBS protocol starting with DNA extraction from frozen rat tissues. We describe steps for RRBS library preparation, library quality control, and sequencing. We also detail an optimized pipeline for sequencing data processing. This protocol has been applied successfully to DNAme profiling across multiple rat tissues.

For complete details on the use and execution of this protocol, please refer to Nair et al.^1^

## Highlights

• Steps for extracting genomic DNA from cryopulverized rat tissues
• An automated protocol for high-throughput RRBS library preparation
• A pipeline for processing the RRBS library sequencing data
• Quality control steps for assessing DNA, libraries, and sequencing datasets

## Abstract (editor highlights)

Publisher’s note: Undertaking any experimental protocol requires adherence to local institutional guidelines for laboratory safety and ethics.

## Graphical abstract

**Figure:**
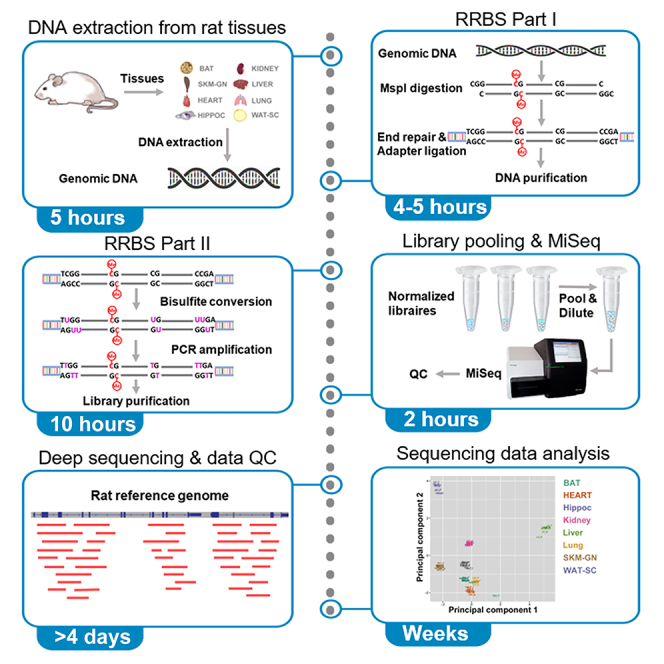

## Before you begin

**Figure 1:**
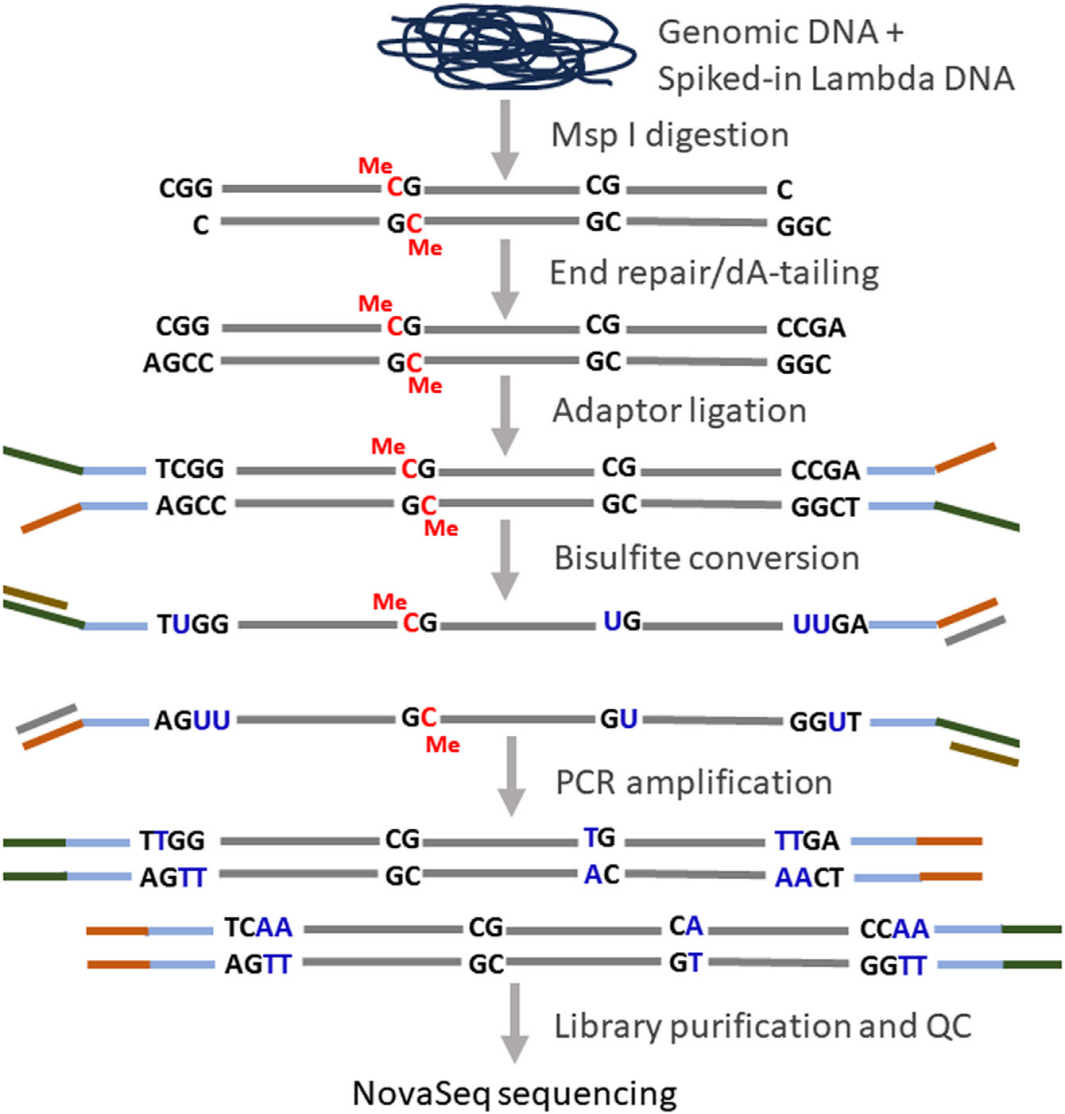
Principle of the RRBS method

RRBS allows the mapping of DNA methylation across the genome at single-nucleotide resolution. Focusing on CpG-rich genomic regions known as CpG islands, this method targets the majority of gene promoters, where CpG islands are primarily found,^2^ thus reducing sequencing requirements and costs compared to whole genome bisulfite sequencing.^3^ RRBS relies on DNA digestion by the methylation-insensitive restriction enzyme MspI, end-repair, A-tailing, and ligation to T-tailed and methylated adaptors; DNA fragments are then size-selected, subjected to bisulfite conversion, PCR amplified, and end-sequenced on an Illumina next-generation sequencing (NGS) platform (Figure 1).

**Figure 2:**
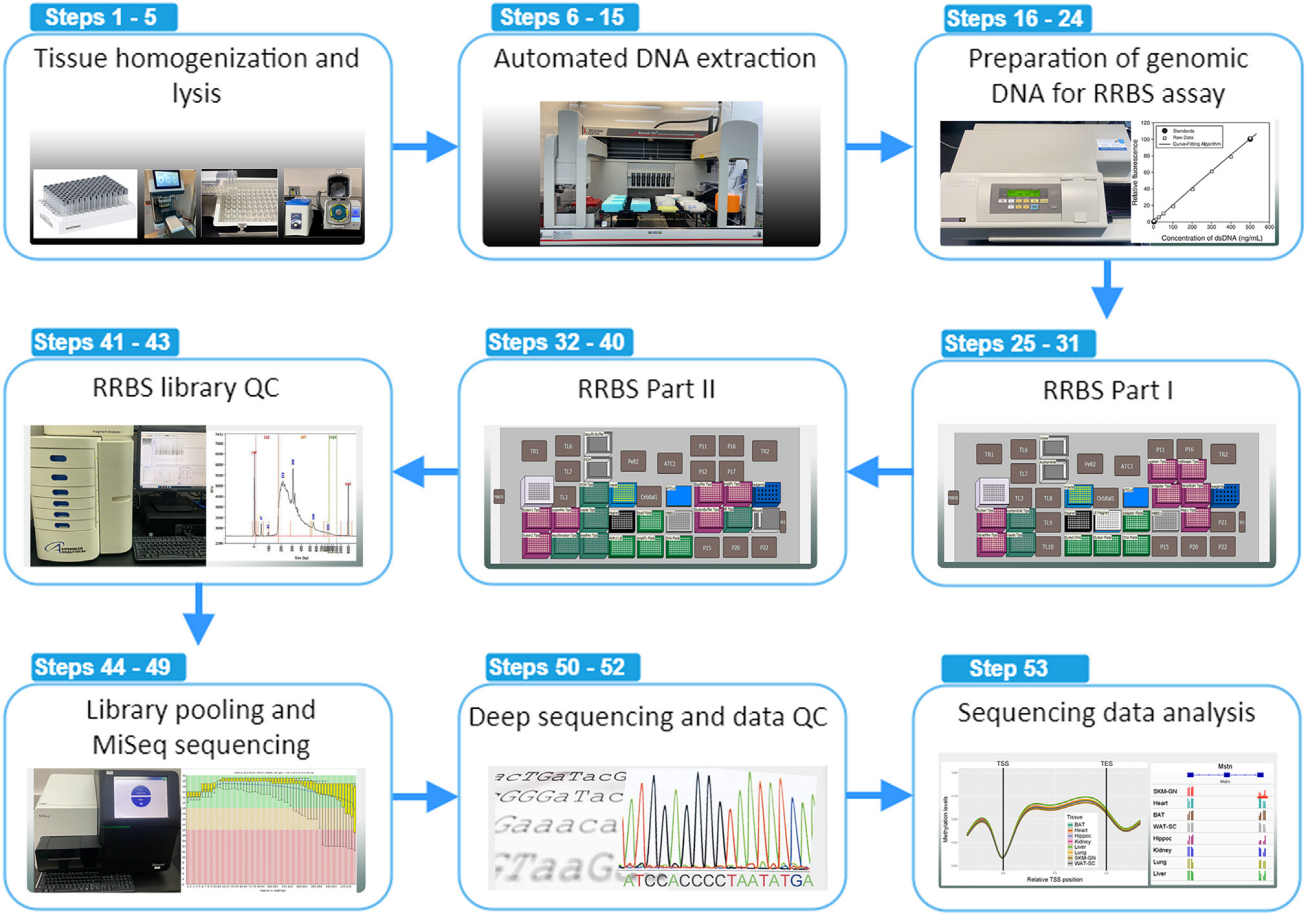
Flowchart of the RRBS protocol Depicted are the major steps of the protocol, from genomic DNA extraction to data analysis.

Herein, we describe the specific steps involved in our automated RRBS procedure, starting with the prerequisite extraction of DNA from 8 frozen rat tissues (gastrocnemius muscle, heart, kidney, liver, lung, hippocampus, subcutaneous white and brown adipose tissues), the RRBS protocol per se, and concluding with data analysis. The main stages of the RRBS procedure are: 1) Tissue homogenization and lysis, 2) Automated DNA extraction, 3) Preparation of DNA for RRBS assay, 4) Automated RRBS library preparation - Part I, 5) Automated RRBS library preparation - Part II, 6) RRBS library quality control (QC), 7) Library pooling and MiSeq sequencing, 8) Deep sequencing and data QC, 9) Sequencing data analysis (Figure 2). While we depict the application of this protocol to rat tissues, it has been successfully tested on snap-frozen human specimens (blood, adipose, and skeletal muscle). Thus, it should be applicable to other species or tissue types.

### Institutional permissions

The Institutional Animal Care and Use Committee at the University of Iowa approved the animal procedures used in the study. As a reminder, users will need to acquire permissions from their own institutions.

### Preparation for DNA extraction

**Timing: 30 min**

Although the steps within the automated DNA extraction protocol have been optimized for the Biomek FX^P^ instrument, they can in principle be adapted for the Biomek i7 or any other automated processing instrument. Before starting the DNA extraction, the experimenter should ensure the availability of the following materials and equipment, as well as the implementation of the ensuing tasks:

1. Ensure that the DNA extraction reagents (see ‘GenFind V3 Kit’ in the key resources table) are in sufficient quantity for the number of samples to process (maximum 96; 80 in this example).
2. Verify that the necessary equipment is available for both the tissue homogenization and the automated DNA extraction (see details in the materials and equipment section).
3. Place tissue homogenizer carriage on ice and Micronic 96-3 Rack on dry ice to ensure that tissue samples will remain cold.
4. Set the water bath temperature to 55°C.
5. Start the computer linked to the Biomek FX^p^ workstation (Beckman Coulter), then turn on the instrument.
6. Open the Biomek software and home all axes of the Biomek FX^p^. Allow the machine to prime the Biomek pipette syringes for approximately 2 min before starting the GenFind DNA isolation protocol.
7. Prepare the following reagents:
  a. Proteinase K-Lysis Buffer mixture:
    i. For each sample, combine 325 μL of Lysis Buffer with 30 μL Proteinase K.
    ii. Calculate the volumes needed based on the number of samples + 10%. For example, for 80 samples, combine (88 × 325 μL) of Lysis Buffer with (88 × 30 μL) of Proteinase K. Mix gently to minimize bubbles.
  b. Assemble the Wash WBC Buffer following the manufacturer’s instructions in the Protocol for GenFind V3 Genomic DNA Isolation Kit (see ‘sample preparation’).
  c. Equilibrate Bind BBB Buffer (magnetic beads) to 15°C–25°C.

***Note:*** Bind BBB Buffer is stored at 4°C. For the best results, we recommend using Wash WBC Buffer within 6 months of assembly (i.e. the addition of Ethanol).

### Preparation for RRBS library construction

**Timing: 15 min**

While the steps within Part I and Part II of the RRBS Library preparation have been optimized for the Biomek i7 instrument, they can in principle be adapted for any other automated processing instrument. Before starting the 2-day procedure (one day for Part I, one day for Part II), the experimenter should ensure the availability of the following materials and equipment, as well as the implementation of the ensuing tasks:

8. For the best results, ensure that two continuous days can be allocated to performing the full RRBS Library preparation procedure.
9. Ensure that the DNA is normalized to 11.8 ng/μL in 8.5 μL (100 ng) to start the library preparation procedure (refer to the DNA quality control and dilution step).
10. Ensure that the RRBS reagents (see ‘Ovation RRBS Methyl-Seq System with TrueMethyl oxBS, 96 reactions’- in the key resources table) are in sufficient quantity for the number of samples to process (80 in this example).
11. Ensure that the necessary equipment is available as needed to set up the Biomek i7 for both Part I and Part II of the RRBS library preparation (see details in the materials and equipment section).
12. Start the computer linked to Biomek i7 and turn on the instrument.
13. Open the Biomek 5 software and home all axes of the Biomek i7. Allow the machine to prime the Biomek pipette syringes by running for approximately 2 min before starting the protocol.
14. Turn on the Pelt1 module and the automated thermal cycler (ATC) control box on the Biomek instrument. Initialize the ATC to establish connection between the Biomek computer and the ATC.

***Note:*** The use of a humidifier near the Biomek i7 while running the RRBS Library preparation procedure is recommended to prevent potential evaporation of sample and reagent volume over the course of the procedure.

## Key resources table

**Table:** 

REAGENT or RESOURCE	SOURCE	IDENTIFIER
**Biological samples**		

Gastrocnemius muscle rat tissue	MoTrPAC Study Group 2022^4^	GEO: GSE242358
Heart rat tissue	MoTrPAC Study Group 2022^4^	GEO: GSE242358
Kidney rat tissue	MoTrPAC Study Group 2022^4^	GEO: GSE242358
Liver rat tissue	MoTrPAC Study Group 2022^4^	GEO: GSE242358
Lung rat tissue	MoTrPAC Study Group 2022^4^	GEO: GSE242358
Hippocampus rat tissue	MoTrPAC Study Group 2022^4^	GEO: GSE242358
Subcutaneous white adipose rat tissue	MoTrPAC Study Group 2022^4^	GEO: GSE242358
Subcutaneous brown adipose rat tissue	MoTrPAC Study Group 2022^4^	GEO: GSE242358

**Chemicals, peptides, and recombinant proteins**		

100% ethanol	Thermo Fisher Scientific	BP2818500
HPLC grade 100% acetonitrile	Thermo Fisher Scientific	A998-1
1x PBS	Thermo Fisher Scientific	10010023
20X TE buffer (200 mM Tris-HCL, 20 mM EDTA, pH 7.5)	Thermo Fisher Scientific	R11490
Low-EDTA TE buffer (10 mM Tris, 20 mM EDTA, pH 8.0)	Thermo Fisher Scientific	J75793-AE
UltraPure DNase/RNase-free distilled water	Invitrogen	10977015
Unmethylated Lambda DNA	Promega	D1521
PhiX control v3	Illumina Inc.	FC-110-3001

**Critical commercial assays**		

GenFind V3 kit – 384 preps	Beckman Coulter	C34881
Qubit dsDNA quantitation, high sensitivity assay (Qubit dsDNA HS assay kit, 500 assays)	Thermo Fisher Scientific	Q32854
Qubit dsDNA quantitation, broad range assay (Qubit dsDNA BR assay kit, 500 assays)	Thermo Fisher Scientific	Q32853
Ovation RRBS Methyl-Seq system with TrueMethyl oxBS, 96 reactions	Tecan	9522-A01
MiSeq reagent kit v2 (300-cycles)	Illumina Inc.	MS-102-2002
AMPure XP beads	Beckman Coulter	A63881
NovaSeq 6000 S4 reagent kit v1.5 (200 cycles)	Illumina Inc.	20028313
High sensitivity NGS fragment analysis kit (1 bp–6,000 bp), 500 samples	Agilent Technologies	DNF-474-0500

**Deposited data**		

MoTrPAC raw and processed data	MoTrPAC Study Group 2022^4^	GEO: GSE242358
MoTrPAC processed data and analysis results	MoTrPAC Study Group 2022^4^	Zenodo: 10099329; GitHub: MoTrPAC/MotrpacRatTraining6moData
Study analysis results	Nair et al., 2024^1^	https://doi.org/10.5281/zenodo.7199920; https://zenodo.org/deposit/7199920

**Experimental models: Organisms/strains**		

Fischer 344 (F344), inbred rat strain, 8 months of age, males and females	National Institute of Aging (NIA)	RRID: RGD_734478

**Software and algorithms**		

bcl2fastq2 Conversion Software v2.20	Illumina	https://support.illumina.com/downloads/bcl2fastq-conversion-software-v2-20.html
RRBS data processing pipeline	MoTrPAC Study Group 2022^4^	Zenodo: 7853676; GitHub: MoTrPAC/motrpac-rrbs-pipeline
R package RnBeads v2.0.	Müller et al. 2019^5^	https://rnbeads.org/

**Other**		

10 mL screw cap tube, internal V-bottom, bulk	Micronic	1775-2318
Polypropylene screw cap with silicone O-ring for Micronic tubes with internal threads, white, 960/pack	Micronic	1775-3101
Micronic 96-3 rack with high cover for tubes capped with screw caps (barcoded A1-H1 side), 10 racks	Micronic	1775-1208
Micronic screw cap recapper CS700	Micronic	CS700
Bead Ruptor Elite tissue homogenizer (4896 sample capacity)	Omni International	SKU 19-040E
LabTie "Gravity" bead loader, 96-well bead dispenser, 1–2.8 mm bead	LabTIE	LT-96-2-1-28
2.8 mm ceramic beads bulk, 325 g	Omni International	SKU 19-646
Tracxer code reader, RS 210	Micronic	MP55120
Biomek FX^p^ automated workstation	Beckman Coulter	A31844
Biomek i7 automated workstation	Beckman Coulter	B87585
Biomek AP96 tips, P250 sterile barrier	Beckman Coulter	717253
Biomek P1000-span-8 tips, sterile barrier	Beckman Coulter	B01124
Biomek i-series 50 μL tips, sterile filtered	Beckman Coulter	B85888
Biomek i-series 190 μL tips, sterile filtered	Beckman Coulter	B85911
Finnpipette Novus multichannel pipette, 8-channel, 100–1,200 μL	Thermo Fisher Scientific	46300800
epDualfilter T.I.P.S. 20–300 μL PCR clean/sterile	Eppendorf	0030 078 560
TempPlate EXT sealing foil	USA Scientific	2988-7100
Microseal 'B' adhesive seals, pack of 100	Bio-Rad	MSB1001
2-mL 96-square DeepWell microplate	Thermo Fisher Scientific	NC1557925
96-well low-profile PCR plate, skirted (black lettering, yellow)	Thermo Fisher Scientific	AB0800YL
Hard-shell low-profile thin-wall 96-well skirted PCR plate	Bio-Rad	HSP9601
96-well clear V-bottom 450 μL polypropylene deep well plate, sterile	Axygen, Corning Life Sciences	P-96-450V-C-S
Fisherbrand 96-well DeepWell polypropylene microplate, sterile	Thermo Fisher Scientific	12-566-611
Eppendorf twin.tec PCR plate 96 semi-skirted, 250 μL	Eppendorf	951020303
ViewPlate-96 black, optically clear bottom, tissue culture treated, sterile, 96-well w/lid (PerkinElmer 6005182)	Thermo Fisher Scientific	50-905-1605
SpectraMax microplate reader, model M2 or higher	Molecular Devices	M2
SoftMax Pro software for SpectraMax, ver. 7.0 or higher	Molecular Devices	SoftMax Pro 7.0
NanoDrop One spectrophotometer (pedestal position only)	Thermo Fisher Scientific	ND-ONE-W
Fragment Analyzer, model 5300 or higher	Agilent	5300
Fragment Analyzer software, version 4.0.0.11 or higher	Agilent	4.0.0.11
ProSize analysis software, version 5.0.1.3 or higher	Agilent	5.0.1.3
CELLTREAT Scientific Products 250 mL conical bottom centrifuge tube, sterile, 48/pack	Thermo Fisher Scientific	50-111-7985
Bioanalyzer, model 2100 or higher	Agilent	2100 or higher
Bioanalyzer software, version B.02.08.SI648 or higher	Agilent Technologies	B.02.08.SI648 or higher
High sensitivity DNA Bioanalyzer kit	Agilent Technologies	5067-4626
Microcentrifuge	Benchmark Scientific	C1012
Water bath	VWR	WB05
Biomek stand, reservoir	Beckman Coulter	372795
Biomek tube block	Beckman Coulter	A83054
12-channel reservoir (trough)	Axygen, Corning Life Sciences	RES-MW12-HP
Quarter reservoir sterile	Beckman Coulter	372790
96S super magnet plate	Alpaqua Engineering, LLC A001322 / Thermo Fisher Scientific	NC0284961
Auto-sealing metal plate lid for thermal cycler, reusable sealer	Bio-Rad	MSL2022
2-mL conical screw cap tubes	USA Scientific	1420-8710
Mixer HC thermomixer	USA Scientific	8012-0000
NovaSeq 6000 system (sequencer)	Illumina Inc.	NovaSeq 6000
MiSeq system (sequencer)	Illumina Inc.	MiSeq
Dry-ice/ice container	Fisher Scientific	07-210-123

## Materials and equipment

• Equipment setup for tissue homogenization and automated DNA extraction:
  ○ Dry ice and a dry-ice/ice container (Fisher Scientific, 07-210-123).
  ○ Ice and an ice container.
  ○ Tissue homogenizer (Bead Ruptor Elite, Omni International, SKU 19-040E).
  ○ Water bath (VWR, WB05).
  ○ LabTie Gravity Bead Loader (LabTIE, LT-96-2-1-28).
  ○ 2.8-mm ceramic beads (Omni International, SKU 19–646).
  ○ Biomek FX^p^ automated workstation (Beckman Coulter, A31844).
  ○ 96-well format magnetic separator (96S Super Magnet Plate, Thermo Scientific, NC0284961).
  ○ 96-square deep well plate (Fisher Scientific, NC1557925): for the lysed tissue samples.
  ○ 96-well PCR plate (Thermo Scientific, AB0800YL): for the eluted DNA (DNA extraction).
  ○ Quarter module reagent reservoir (Beckman Coulter, 372790): for the BBB Buffer; water for the DNA dilution.
  ○ 12-channel high-profile reagent reservoirs (Axygen, RES-MW12-HP): for the Wash WBB and WBC Buffers, and the Elution Buffer.
  ○ P1000 and P250 pipette tips (Beckman Coulter, B01124 and 717253, respectively) for Biomek FX^p^ and for 8-channel pipette.
  ○ Micronic 96-3 Rack (Micronic, 1775-1208).
• Equipment setup for RRBS Parts I and II:
  ○ Ice and an ice container.
  ○ Biomek i7 automated workstation, equipped with an on-deck thermal cycler and reagent cooling block (Beckman Coulter, B87585).
  ○ 2.0-mL conical screw cap tubes (USA Scientific, #1420–8710): for the MspI, Ligation, and Final Repair Master Mixes, and for the Denaturing Solution.
  ○ 12-channel high-profile reagent reservoirs: for the 80% Acetonitrile, Nuclease-free water, 70% Ethanol, and Desulfonation Buffer
  ○ Biomek i-series 50 μL Sterile Filtered Tips (Beckman Coulter, B85888).
  ○ Biomek i-series 190 μL Sterile Filtered Tips, (Beckman Coulter, B85911).
  ○ Hard-shell thin-well 96-well PCR plates (Bio-Rad, HSP9601): for the Denatured DNA (RRBS Part I) and the purified, Bisulfite-converted DNA (RRBS Part II).
  ○ 96-well PCR plate (Thermo Scientific, AB0800YL): for the purified amplified RRBS libraries.
  ○ Quarter module reagent reservoir: for MBBS1, MBBS2, and AMPure XP Beads.
  ○ 96 Well Clear V-Bottom 450 μL Polypropylene Sterile Deep Well Plate (Axygen, P-96-450V-C-S): for MBBS1 and MBBS2.
  ○ Unmethylated Lambda DNA (Promega, D1521).
  ○ Heated orbital incubator ‘Mixer HC’ (USA Scientific, 8012-0000): for the Bisulfite Reagent Solution (RRBS Part II).

## Step-by-step method details

### DNA extraction

**Timing: 3 h**

Genomic DNA is extracted from cryopulverized rat tissues using a high-throughput, semi-automated method that is adapted from the protocol (downloadable from Zenodo: 10656069), for GenFind V3 Genomic DNA Isolation Kit (Beckman Coulter, Indianapolis, IN). The procedure consists of seven major steps: tissue homogenization and lysis, binding of the genomic DNA to magnetic beads, separation of the beads from contaminants, two consecutive washes of the beads to remove contaminants, resuspension of the beads in elution buffer, and separation of genomic DNA from the beads. While tissue homogenization and lysis are performed manually and with a tissue homogenizer (Steps 1–5), the remainder of the DNA extraction is carried out in an automated fashion on a Biomek FX^p^ workstation (Steps 6–15).

***Note:*** Cryopulverized tissue samples are previously stored at −80°C in Micronic tubes that are held in a Micronic rack. Tissue aliquots of 10–30 mg (15 mg for white adipose tissue and 30 mg for brown adipose tissue) are used for DNA extraction.

**Figure 3:**
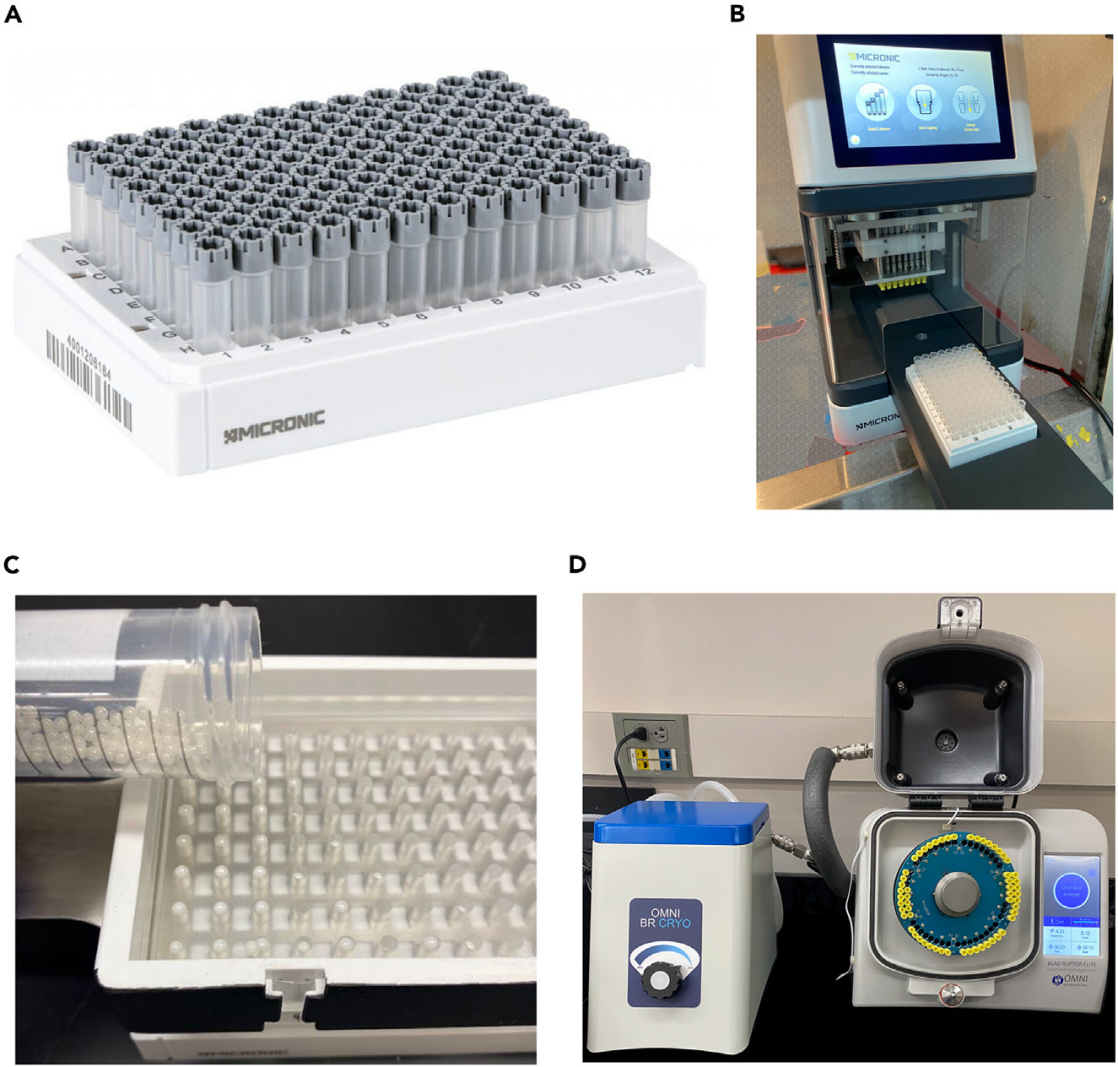
Illustration of key sub-steps of tissue homogenization (A) Image of the Micronic rack holding the Micronic tubes that contain frozen tissue aliquots. (B) Image of the decapping of Micronic tubes using a Recapper. (C) Image of the pouring of ceramic beads onto the bead loading tool that is designed to load the Micronic tubes with ceramic beads. (D) Image of the sealed Micronic tubes loaded onto the chilled tissue homogenizer carriage. The Micronic tubes contain tissue aliquots, ceramic beads, and the Proteinase K-Lysis Buffer mixture.

***Note:*** Avoid thawing the tissue samples before the subsequent addition of Proteinase K-Lysis Buffer mixture at Step 3.

**Figure 4:**
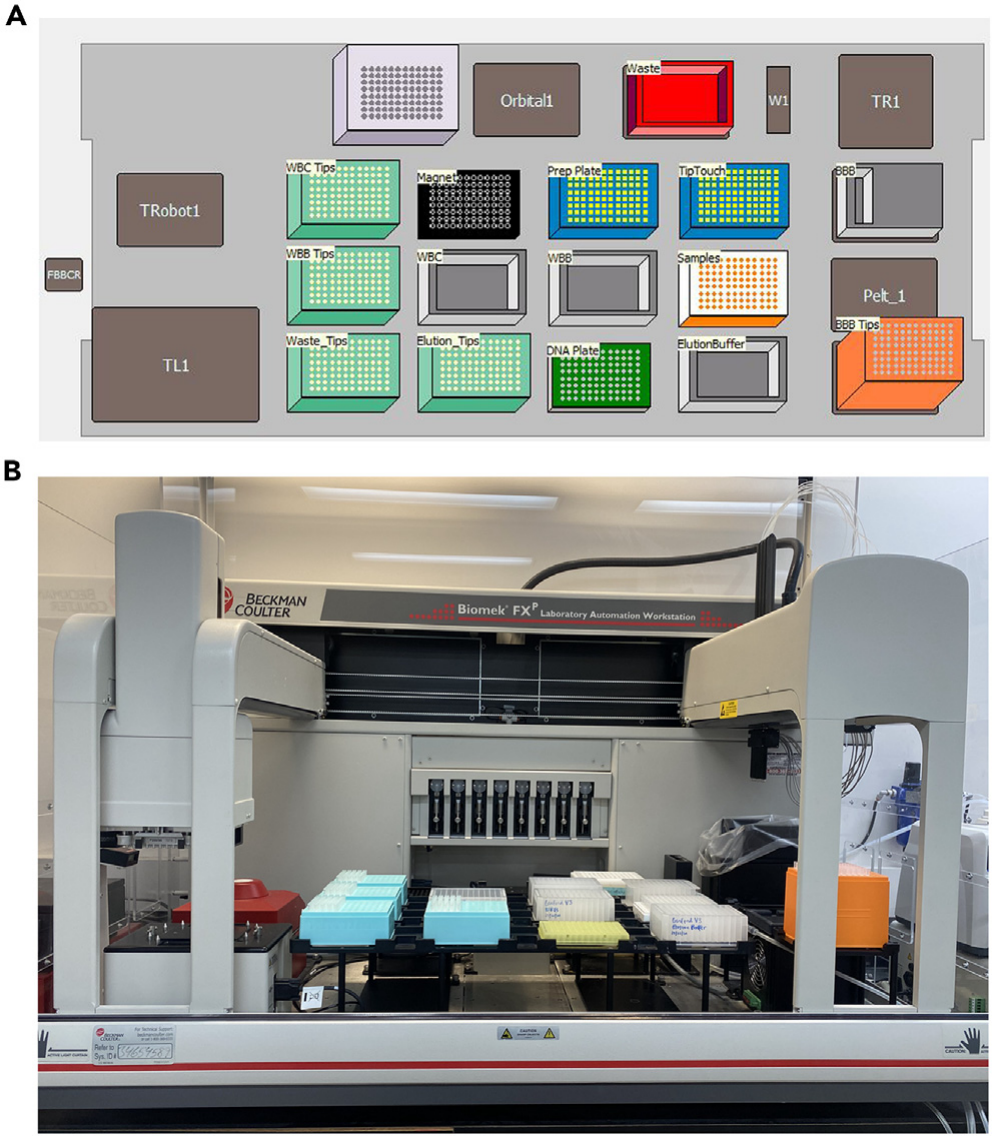
The Biomek FX^p^ instrument setup used for automated genomic DNA extraction from rat tissues (A) Biomek FX^p^ deck layout for the GenFind V3 protocol, as shown on the Biomek-linked computer. (B) Image of the Biomek FX^p^ deck set up for the GenFind V3 protocol, with consumables. Orbital1, orbital shaker; W1, wash station position 1 (for the span-8 pipettor of the instrument); TR1, trash receptacle position 1 (for the instrument to dispose of tips); TL1, tip loading position 1 (where tips are loaded onto the instrument 96-multichannel); Trobot1, FBBCR, and Pelt_1 are not used in the experiment.
  b. Fill 12-channel reagent reservoirs with the appropriate volumes of Agencourt GenFind Wash WBB and WBC Buffers, and Elution Buffer (UltraPure water, Invitrogen, 10977015).

1. Transfer the Micronic rack holding the Micronic tubes (Figure 3A) of frozen tissue aliquots from −80°C storage to dry ice to prepare for DNA isolation.

2. Load the Micronic rack into the Micronic Screw Cap Recapper and decap the Micronic tubes (Figure 3B).
3. Proceed with the Homogenization and Lysis of the tissues.
  a. Using an 8-channel pipette, add 355 μL of Proteinase K-Lysis Buffer mixture (refer to the preparation for DNA extraction section, Step 7a) to each sample/Micronic tube.
  b. Add three 2.8-mm ceramic beads to each sample/Micronic tube:
    i. On the Micronic rack, add empty uncapped Micronic tubes to fill in the empty positions on the 96-well rack.
    ii. Carefully attach the bead loading tool to the Micronic tubes. Ensure that the loading tool is aligned with all 96 Micronic tubes on all sides of the Micronic rack.
    iii. Ensure that the bead loading tool is in the closed position. Carefully pour a 50-mL tube of ceramic beads onto the loading tool (Figure 3C). Using sterilized forceps or a pipette tip, fill in each of the 96 holes of the loading tool with 3 ceramic beads.
    iv. When the loading tool is properly filled with 3 beads per well position, slide open the loading tool to load the Micronic tubes with the ceramic beads.
    v. Remove the empty tubes with ceramic beads. Clean empty tubes and unused ceramic beads can be put away for another use.
  c. Add 125 μL of 1x PBS to each sample.
  d. Load the Micronic rack into the Micronic Screw Cap Recapper and recap the Micronic tubes.
  e. Load the sealed Micronic tubes onto the chilled tissue homogenizer carriage (refer to the preparation for DNA extraction section, Step 3), taking care to balance the carriage so the sample load is evenly distributed.
  f. Place the balanced tissue homogenizer carriage into the homogenizer and secure it tightly (Figure 3D). Input settings of 4.2 speed (m/s), 2 Cycles, 00:20 Time, and 00:10 Dwell. Run the homogenizer.
  g. When homogenization is complete, place the homogenizer carriage on ice and transfer the tubes into the Micronic rack, also on ice.
  h. Incubate the Micronic tubes in a 55°C water bath for 30 min.
4. During the 55°C incubation:
  a. Open the GenFind V3 protocol file, VN_GenFind v3_Tissue.bmf (downloadable from Zenodo: 10656069), on the Biomek-linked computer and set up the Biomek FX^p^ deck layout accordingly (Figures 4A and 4B).

VolumeofWBBBufferfor80samples=88×600μL=52.8mL

VolumeofWBCBufferfor80samples=88×900μL=79.2mL

VolumeofUltraPurewaterfor80samples=88×115μL=10.1mL

***Note:*** When handling a 12-channel reagent reservoir, each channel is used to dispense reagent into a column (8 samples/8 wells) of the 96-well plate. We recommend adding an extra 2-mL to the amount of buffer needed per channel, so as to account for the residual volume left in each channel, and ensure that the Biomek instrument is able to aspirate the full volume needed.

5. After the 55°C incubation, remove the Micronic rack from the water bath and dry the rack with paper towels as needed. Load the Micronic rack into the Micronic Recapper and decap Micronic tubes.
6. Place the Micronic rack holding the samples on the Biomek FX^p^ deck in accordance with the deck layout (Figure 4A).
7. Right before starting the Biomek FX^p^ procedure, vortex the BBB buffer bottle to ensure the solution is evenly mixed. Fill a Quarter module reagent reservoir with the appropriate volume of BBB Buffer.

Volumefor80samples=88×201μL=17.7mLBBBBuffer

8. Start the Biomek Genfind V3 protocol.

***Note:*** The experimenter should follow a series of dialog prompts for: i) inputting the number of samples in multiples of 8 and ii) confirming the Biomek deck layout.

**CRITICAL:** Ensure that all labware and reagents are placed on the deck layout correctly (Figure 4). Failure to verify that the Biomek instrument deck is set up exactly as shown in the protocol deck layout may result in instrument collisions or experimental issues such as improper tip wash after waste removals, leading to sample contamination and reduced purity.

***Note:*** P250 tips are used throughout the protocol, except for dispensing the Bind BBB Buffer (beads), which is done using P1000 tips.

9. Clamp tubing for Biomek wash station.
10. Using the Biomek FX^P^, transfer 400 μL of lysed tissue samples from Micronic tubes to a 96-Square deep-well plate. Once samples have been transferred, the Micronic rack can be removed from the deck layout and loaded onto the Micronic recapper to recap the tubes.

***Note:*** Steps 11–14 below are similar to the protocol for GenFind V3 Genomic DNA Isolation Kit, except that several volumes, incubation times, and the supernatant aspiration technique were modified to optimize the automated DNA extraction.

11. Proceed with the addition of Bind BBB Buffer (magnetic beads) following the manufacturer’s instructions (Procedure, steps 4–7), with the following modifications:
  a. At step 4a, add 201 μL of Bind BBB Buffer per sample well.
  b. At step 5, incubate the sample plate at 20°C–25°C for 10 min.
  c. At steps 6–7, place the plate on the magnetic separator for 15 min, then pick up and discard the supernatant in successive 150-μL aliquots while the plate is on the magnetic separator.

***Note:*** If beads are being aspirated during the transfer, dispense the sample back into the well and let the plate sit for an additional 10 min to better compact the beads.

12. Proceed with the WBB washes following the manufacturer’s instructions (Procedure, steps 8–11), with the following modifications:
  a. At step 8a, dispense 150 μL of Wash WBB Buffer 4 consecutive times (600 μL in total).
    ***Note:*** Ensure that beads are completely resuspended during wash steps. During every lysate/wash removal, check to confirm that no beads are seen within the supernatant being removed from the sample plate.
  b. At step 9, incubate the sample plate at 20°C–25°C for 2 min before placing it on the magnetic separator for 10 min.
  c. At step 10, pick up and discard the supernatant in successive 150-μL aliquots, while the plate is on the magnetic separator.
13. Proceed with the WBC washes following the manufacturer’s instructions (Procedure, steps 12–15), with the following modifications:
  a. At step 12, dispense 150 μL of Wash WBC Buffer 6 consecutive times (900 μL in total).
  b. Incubate the sample plate at 20°C–25°C for 2 min before placing it on the magnetic separator for 7 min (step 13).
  c. At step 14, pick up and discard the supernatant in successive 150-μL aliquots, while the plate is on the magnetic separator.

**CRITICAL:** Do not let the beads over-dry and crack.

14. Proceed with the Elution following the manufacturer’s instructions (Procedure, steps 16–18), with the following modifications:
  a. At step 16, add 115 μL of UltraPure water to each sample well.
  b. At step 18, place the plate on the magnet for 10 min. Transfer 100 μL of the supernatant to a 96-well PCR plate (Thermo Scientific, AB0800YL). The eluted genomic DNA is suitable for DNA quality control.

**Pause point:** The experimenter can pause after elution of the DNA. The plate of eluted DNA can be sealed with a foil seal (USA Scientific, 2988–7100) and stored at −20°C.

15. Unclamp Biomek tubing for the wash station.
  a. Dispose of leftover reagents/waste.
  b. Wash reagent reservoirs with deionized water.
  c. Clean up the Biomek waste tray and tubing with diluted bleach.
  d. Wash out using deionized water.

### DNA quality control and dilution

**Timing: 75 min**

Following DNA extraction, eluted DNA samples are assessed qualitatively and quantitatively, and subsequently diluted to 11.8 ng/μL. Qualitative assessment is done by measuring the ratio of absorbance at 260 nm and 280 nm (A260/280) and the ratio of absorbance at 260 nm and 230 nm (A260/230) on a NanoDrop One spectrophotometer (Thermo Fisher Scientific, ND-ONE-W). Both ratios are used as measures of nucleic acid purity. For the quantification of DNA, the Qubit dsDNA Assay Kit (Thermo Fisher Scientific, Q32854) is used, as it is highly selective for double-stranded DNA (dsDNA) vs. single-stranded DNA, RNA, and free nucleotides. The kit contains a fluorescent dye that selectively binds to DNA, and the fluorescence signal is measured on a microplate reader. Thus, when it comes to determining DNA concentration, Qubit is more accurate than NanoDrop.

16. Evaluate DNA quality on the NanoDrop spectrophotometer:
  a. Randomly select one sample well per column in the Eluted DNA plate, and measure 1 μL of stock DNA sample to spot-check concentration/purity.
  b. Measure more samples from the plate as needed. Problem 1.

***Note:*** A 260/280 ratio of ∼1.8 is indicative of “pure” DNA, whereas a lower ratio denotes the presence of protein, phenol, or other contaminants that absorb strongly at 280 nm. The 260/230 ratio is also indicative of DNA purity, with values expected to be in the 2.0–2.2 range. A significantly lower 260/230 ratio signifies the presence of contaminants such as phenol and guanidine HCl. Contamination of the DNA may result in an overestimation of its concentration, and subsequently in a lower signal in the assay.

17. Quantify DNA with Qubit assay, i.e., using either the Qubit dsDNA High Sensitivity Assay Kit (Thermo Fisher Scientific, Q32854) or the Qubit dsDNA Broad Range Assay Kit (Q32853), as specified below:
  a. Turn on the SpectraMax microplate reader (Molecular Devices, M2) and computer connected to the instrument. Open the SoftMax Pro software.
  b. Depending on your NanoDrop concentrations, decide which Qubit assay and range you will use for your measurement.
    ***Note:*** Qubit dsDNA Broad Range assay measures DNA concentrations between 0.2-2,000 ng/μL, whereas Qubit dsDNA High Sensitivity assay measures DNA concentrations between either 0.005–120 ng/μL.
    ***Note:*** If your NanoDrop concentrations are above the Qubit ranges indicated above, prepare a separate Diluted DNA sample plate with the appropriate dilution factor to ensure your sample concentrations fall within range. A 3-μL sample volume is needed per Qubit measurement.
  c. Determine DNA concentration using the Qubit Assay in a SpectraMax plate (Fisher Scientific, Cat # 50-905-1605), following the manufacturer’s instructions.
    ***Note:*** If using Qubit Broad Range assay, dilute the 100 ng/μL stock standard using 1x TE buffer to create 8 standards for the broad range curve: 0, 5, 10, 20, 40, 60, 80, 100 ng/μL.
  d. Review the instrument measurements:
    i. Standard curve: an Rˆ2 value of 0.98 or greater is acceptable. If the Rˆ2 value is less than 0.98, the calculated Qubit concentrations may be less accurate, and the Qubit plate may need to be prepared again with a new standard curve for repeated measurements.
    ii. DNA samples: DNA concentrations should fall within the range of the selected standard curve. If they are out of range (marked by “R” on the software), DNA concentrations may be less accurate.
    iii. If DNA samples were diluted for Qubit measurement, multiply the Qubit concentration values by the dilution factor to obtain the DNA stock concentrations.
  e. Once measurements are completed, either dispose of the assay plate or save the unused wells for future SpectraMax measurements. Record the experimental DNA concentration values (ng/μL) by exporting the results as an Excel file. Problem 2.
    **Pause point:** The experimenter can pause after completing the Qubit measurements. DNA can be stored at 4°C for a few days and at −20°C for the long term.
18. To prepare the DNA dilutions, thaw on ice the eluted DNA plate from Step 14.
19. Start the computer linked to the Biomek i7 workstation, then turn on the workstation.
20. Open the Biomek software, home all axes of the Biomek i7, and allow the machine to prime the Biomek pipette syringes for approximately 2 min.
21. Open the Dilution protocol file, Dilution Template Example.csv (downloadable from Zenodo: 10656069), on Biomek i7 and set up the deck layout accordingly. The layout comprises a Diluted DNA plate (Thermo Scientific, AB0800YL), a Diluted DNA Aliquot plate (Bio-Rad, HSP9601), a Quarter Module Reservoir containing Nuclease-free water (provided in the RRBS kit) or UltraPure water for sample dilution, and any Biomek tips as needed for pipetting dilution volumes.
22. Prepare the dilution file with the dilution calculations for each DNA sample and import it into the Biomek dilution procedure:
  a. Choose a fixed Sample volume and calculate the Water volume needed to prepare an 11.8 ng/μL dilution, using the Ci × Vi = Cf × Vf equation, where Ci = Qubit concentration, Vi = Sample volume, Cf = 11.8 ng/μL, Vf = Final Dilution volume. The volume of water needed for the dilution will be Vf-Vi.
  b. Enter the Water volume needed per sample well into the dilution file and save the file as .csv. Ensure that the calculated volumes are within the pipetting volume range of the Biomek tips that will be used for the dilution procedure. Increase the pipette tip size or decrease the amount of DNA sample volume used for dilution as needed.
  c. Upload the .csv dilution file to the Dilution protocol file on Biomek i7.

***Note:*** The Biomek procedure is written to add a fixed volume of DNA to every well in the sample plate. The volume of water needed will vary based on the Qubit DNA concentrations. It is recommended to prepare a dilution plate of at least 20 μL, so the dilution plate has sufficient volume to repeat RRBS library prep if needed, without the need of creating another sample dilution from the stock DNA plate.

23. Run the Biomek DNA dilution protocol.
  a. Input the volume of DNA that will be added for each sample into the dilution plate.
  b. Input the mixing volume for the dilution plate to ensure that the sample dilutions are properly mixed.
  c. Verify the consumables and plate locations on the Biomek deck layout before starting the procedure.
  d. The protocol consecutively generates a Diluted DNA plate (11.8 ng/μL) and a Diluted DNA Aliquot plate containing at least 8.5-μL per 11.8 ng/μL diluted sample (= 100 ng) from the Diluted DNA plate.
24. Once DNA dilution has been completed, seal both plates securely and store both dilution plates at −20°C until ready for use in RRBS library preparation.

**Pause point:** The experimenter can pause after diluting the DNA. Diluted DNA remains stable at −20°C for several months.

### RRBS part I - MspI digestion, adaptor ligation, final DNA end repair, DNA purification and denaturation

**Timing: 4-4.5 h**

*The RRBS Library preparation is performed in an automated fashion on the Biomek i7 workstation, following the*Ovation RRBS Methyl-Seq System 1–16*(NuGEN, Catalog #0553) protocol* (downloadable from Zenodo: 10656069)*, with the exception of a few modifications. Part I consists of four steps, namely, MspI digestion, Adaptor ligation, Final DNA end repair, DNA purification and denaturation.*

**Figure 5:**
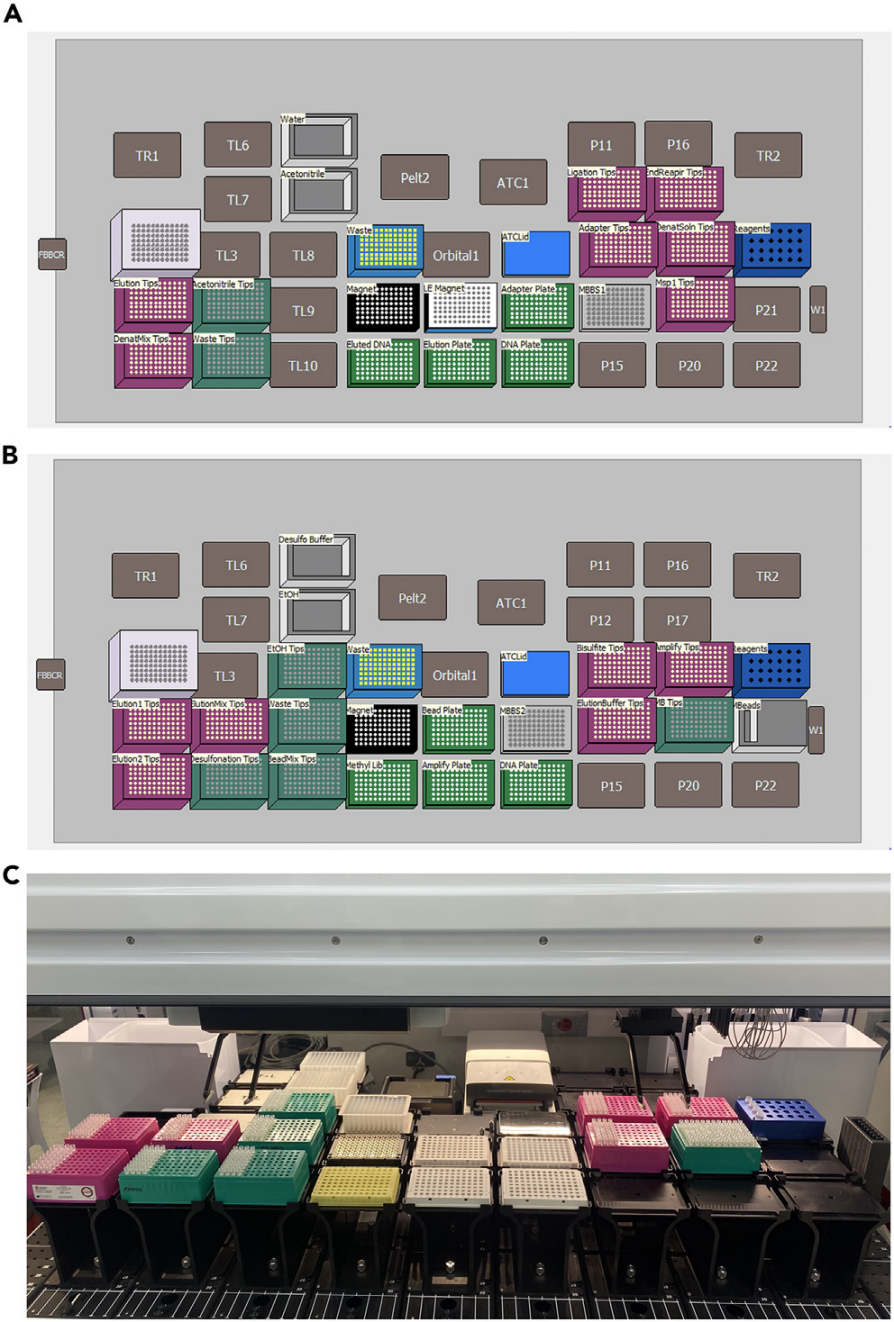
The Biomek i7 instrument setup used for the RRBS protocol (A and B) Biomek i7 deck layouts for RRBS Part I (A) and for RRBS Part II (B), as shown on the Biomek-linked computer. (C) Image of the Biomek i7 deck set up for the RRBS Part II protocol, with consumables. ATC1, automated thermal cycler; TR2, second trash receptacle; TL3, 6, 10, tip loading positions 3, 6, 10; Pelt_2 is not used in the experiment. P11-12, P15-17, and P20-22 are empty positions that are not used in the experiment either.
26. Proceed with the MspI Digestion procedure following the manufacturer’s instructions (Protocol, steps B1-3), with the following modifications at step B3:
  a. Prepare MspI Master Mix by combining MspI Buffer Mix, MspI Enzyme Mix, and Unmethylated Lambda DNA (at an initial concentration of 7 ng/μL):
    Volumefor80samples=88×(1μLBufferMix+0.5μLEnzymeMix+0.0107μLLambdaDNA)
  b. Mix MspI Master Mix by pipetting, then split volume equally into four 2.0-mL conical screw cap tubes, and immediately place the four tubes in the first column of the reagent cooling block on the Biomek i7 workstation.
    ***Note:*** A control is recommended for evaluating the bisulfite conversion efficiency. We recommend the use of Unmethylated Lambda DNA as a positive control for bisulfite conversion. Once it has been diluted to 7 ng/μL with Nuclease-free water, a small amount of diluted Unmethylated Lambda DNA is added to the Mspl Master Mix to ensure that all samples undergoing RRBS Library preparation contain an equal amount of the control DNA. Lambda DNA should be kept on ice until close to starting the procedure.
27. Start the Biomek RRBS Library preparation Part I protocol.

***Note:*** The experimenter should follow a series of dialog prompts for: i) inputting the number of samples in multiples of 8, ii) setting up the addition of Ligation Adaptor Indexes, i.e. indicating which column of the RRBS Adaptor Index plate the Biomek will start from, and iii) confirming the Biomek deck layout.

25. Open the RRBS Part I protocol file, RRBS_Part01_Revised.bmf (downloadable from Zenodo: 10656069), on the Biomek-linked computer, and set up the Biomek i7 deck layout accordingly (Figure 5A).
  a. If the Diluted DNA Aliquot plate is stored at −20°C (see Step 24), thaw it at 20°C–25°C, then place it on the deck layout. This will be the starting sample plate for the RRBS procedure.

28. Using the Biomek i7 workstation, resume the MspI digestion procedure following the manufacturer’s instructions (Protocol, steps B4-5) with the following modification:
  a. At step B5, hold sample plate on deck at 20°C–25°C (instead of 4°C) following MspI digestion in on-deck thermal cycler.
29. Proceed with the Adaptor Ligation procedure following the manufacturer’s instructions (Protocol, C1-7) with the following modifications:
  a. At step C4, add 2 μL of the appropriate Ligation Adaptor Index (instead of 3 μL) to each sample.
  b. At step C5, prepare the Ligation Master Mix as instructed, then split volume equally into four 2.0-mL conical screw cap tubes and immediately place in the second column of the reagent cooling block.
  c. At step C6, add 6.5 μL of the Ligation Master Mix per reaction (instead of 7 μL).
  d. At step C7, hold sample plate on deck at 20°C–25°C (instead of 4°C) following Adaptor ligation in on-deck thermal cycler.
30. Proceed with the Final Repair procedure following the manufacturer’s instructions (Protocol, steps D1-6) with the following modifications:
  a. At step D4, prepare the Final Repair Master Mix as instructed, then split volume equally into four 2.0-mL conical screw cap tubes and immediately place in the third column of the reagent cooling block.
  b. At step D6, hold sample plate on deck at 20°C–25°C (instead of 4°C) following Final repair in on-deck thermal cycler.
31. Proceed with the DNA Purification and Denaturation procedure following the manufacturer’s instructions (Protocol, steps E1-24) with the following modifications:
  a. At step E10, transfer the 50 μL sample from the sample plate into the Axygen deep-well plate containing 100 μL of MBBS1 Master Mix (instead of adding MBBS1 to the 50 μL sample).
  b. At step E12, magnetic separation incubation time is 7 min (instead of 5 min).
  c. At step E17, air dry the bead pellets for 5 min at 20°C–25°C. Simultaneously, split the Denaturing Solution volume equally into four 2.0-mL conical screw cap tubes and immediately place in the reagent block, which should be at 25°C.
  d. At step E19, add 12 μL of Denaturing Solution (instead of 10 μL) onto each bead pellet.
  e. At step E24, transfer 10 μL of the eluate (instead of 9 μL) to a hard-shell thin-well 96-well PCR plate (Bio-Rad). The eluted DNA is suitable for the RRBS Part II protocol.

**Pause point:** The experimenter can pause after elution of the DNA. If pausing for longer than 1 h, the plate of eluted DNA should be sealed tightly with a plastic plate seal (Bio-Rad, MSB1001) and stored at 25°C for 1–24 h in an off-deck thermal cycler, with a thermal cycler lid temperature of 50°C. These storage conditions are essential for preventing sample volume evaporation within a 24-h period.

### RRBS part II - Bisulfite conversion, DNA purification, PCR amplification, Amplified Library Purification

**Timing: 7 h**

RRBS Part II consists of four steps, i.e., Bisulfite conversion of the DNA, Bisulfite-converted DNA desulfonation and purification, Library amplification, and Amplified library purification. Similar to RRBS Part I, Part II is performed in an automated fashion on the Biomek i7 workstation, following the protocol for Ovation RRBS Methyl-Seq System 1–16 (NuGEN, Catalog #0553), with the exception of a few modifications. Of note, the following steps in the NuGEN protocol are omitted: i) DNA Oxidation (optional), as we do not quantify DNA hydroxymethylation (5-hydroxymethylcytosine; 5hmC); ii) Library Amplification Optimization with qPCR, as we have already determined the optimal thermal cycler program for Library Amplification.

***Note:*** If using another organism or other rat sample types, the experimenter may have to optimize the Library Amplification with qPCR.

32. Set the Mixer HC thermomixer (USA Scientific, #8012-0000) on the bench top at 60°C, with a speed of 1000 rpm and a 15-min incubation time.
33. If the eluted DNA plate from Step 31 was stored in a thermal cycler for 1–24 h, remove it from the thermal cycler. Spin down the eluted DNA plate at 200 × *g*, remove the plastic plate seal, and store at 20°C–25°C until ready to load onto the Biomek deck for Part II of the RRBS library preparation.
34. Proceed with the Bisulfite Conversion procedure following the manufacturer’s instructions (Protocol, steps G1-6).
35. Open the RRBS Part II protocol file, RRBS_Part02_Revised.bmf (downloadable from Zenodo: 10656069), on Biomek i7 and set up the deck layout accordingly, including the eluted DNA plate at the completion of Step 31. (Figures 5B and 5C).
36. Start the Biomek RRBS Library preparation Part II protocol.

***Note:*** The experimenter should follow a series of dialog prompts for: i) inputting the number of samples, rounded to the next highest multiple of 8, ii) confirming the Biomek deck layout.

37. Using the Biomek i7 workstation, resume the Bisulfite Conversion procedure as follows:
  a. Add 30 μL of Bisulfite Reagent Solution to each well of the eluted DNA plate, for a total of 40 μL. Mix by pipetting.
  b. Incubate the eluted DNA in the on-deck thermal cycler at 95°C for 5 min, 60°C for 20 min, 95°C for 5 min, 60 °C at 40 min, 95 °C at 5 min, 60 °C at 45 min, then transfer the plate to 20°C–25°C on deck.

**Table 1:** The primary metrics used for assessing the quality of RRBS libraries by the MiSeq system

	Flagged libraries	Passed QC libraries
Read depth	<25% of the median sample depth	High
% of the GC content in trimmed fastq files	20%–30% of the mean per-base GC-content	>20 or <80%
% bisulfite conversion efficiency	<95%	>95%
Duplication rate based on UMI	>20%	<20%
% reads mapped to genome	<50%	>50%
%OT (mapped reads aligned to the original top stand)	<30%	>30%
%OB (mapped reads aligned to the original bottom stand)	>70%	<70%
%CTOT (mapped reads aligned to the complementary to original top strand)	>10%	<10%
%CTOB (mapped reads aligned to the complementary to original bottom strand)	>10%	<10%
FastQC plots∗	Problematic	Good

For reference, see the MoTrPAC GET QC SOP for animal studies (http://study-docs.motrpac-data.org/Animal_GET_QC_SOP.pdf).

∗ FastQC plots comprise ‘Per base sequence quality’ and ‘Per base sequence content’, which plot the Q-score of the raw sequence reads for each cycle and the proportion of each base at each cycle, respectively. The higher ‘Per base sequence quality’ is, the better. In a normal ‘Per base sequence content’, all four bases are equally represented.

**CRITICAL:** The success of the bisulfite conversion is crucial for a successful library amplification. The bisulfite conversion efficiency is evaluated during the MiSeq QC (Step 49; see Table 1).

38. Proceed with the Bisulfite-Converted DNA Desulfonation and Purification procedure following the manufacturer’s instructions (Protocol, steps H1-33), with the following modifications:
  a. At steps H6-7, transfer MBBS2 from a Quarter module reservoir into an Axygen deep well plate (Axygen, Cat. #Corning P-96-450V-C-S) at 160 μL per well and place the plate on the Biomek i7 deck.
  b. At steps H8-10, using the Biomek i7 workstation, transfer 40 μL of the bisulfite-converted DNA to the Axygen deep well plate containing MBBS2, for a total of 200 μL. Mix thoroughly by pipetting and incubate for 5 min at 20°C–25°C.
  c. At step H11, place the plate onto the magnet and incubate at 20°C–25°C for 15 min (instead of 5 min) to completely clear the solution of beads.
  d. At step H12, remove 90 μL of supernatant, then allow incubation for another 2 min to ensure complete magnetic separation. After 2 min, remove another 90 μL of supernatant.
  e. At step H19, after adding the Desulfonation Buffer and pipetting to resuspend the beads, incubate at 20°C–25°C for 5 min, then place the plate onto the magnet and incubate for another 5 min to completely clear the solution of beads.
  f. At steps H30-32, after adding the Elution Buffer and pipetting to resuspend the beads, incubate at 20°C–25°C for 15 min (instead of 5 min). Then, place the plate onto the magnet and incubate at 20°C–25°C for another 15 min (instead of 5 min) to completely clear the solution of beads.
  g. At step H33, transfer 22 μL of the eluate into a Bio-Rad PCR plate.
39. Proceed with the Library Amplification procedure following the manufacturer’s instructions (Protocol, steps J1-6), with the following modifications:
  a. Use AMPure XP Beads (Beckman Coulter, #A63881; formerly called Agencourt Beads).
  b. At step J5, using the Biomek i7 workstation, add 30 μL of Amplification Master Mix to each sample for a total of 52 μL.
  c. At steps J6-7, incubate in on-deck thermal cycler using the PCR cycling conditions provided below, to achieve library amplification.

**Table:** PCR cycling conditions

Steps	Temperature	Time	Cycles
Initial Denaturation	95°C	2 min	1
Denaturation	95°C	15 s	14 Note that the number of cycles should be optimized using qPCR when using the kit for the first time, new sample type, or input.
Annealing	60°C	1 min	
Extension	72°C	30 s	
Hold	10°C	until ready to proceed

40. Proceed with the Amplified Library Purification procedure following the manufacturer’s instructions (Protocol, steps K1-16), with the following modifications:
  a. After steps K1-2, transfer the fully resuspended AMPure XP Beads to a Quarter Module reagent reservoir (Beckman Coulter, #372790), and place it on the Biomek i7 deck.
  b. At step K14, add 27 μL of DR1 (instead of 20 μL) to the dried beads.
  c. At step K16, remove 25 μL of the eluate (instead of 18 μL) and transfer to a fresh PCR plate (Thermo Scientific, AB0800YL).

**Pause point:** Samples may be sealed with a foil seal and stored at −20°C.

### Quality control of the RRBS libraries

**Timing: 2.5 h**

*The quantity of the libraries is measured by*Qubit dsDNA Quantitation, High Sensitivity Assay*(ThermoFisher Scientific), and the quality is evaluated using the*Fragment Analyzer High Sensitivity NGS Fragment Kit*(Agilent Technologies, Santa Clara, CA). Both the Qubit HS assay and Fragment Analyzer analysis are run on the Biomek* FX^p^*. The Qubit HS Assay is highly selective for dsDNA over RNA and is accurate for sample concentrations from 5 pg/μL to 120 ng/μL, providing an assay range of 0.1–120 ng. The Fragment Analyzer results provide the average fragment size in each sample.*

41. Perform the Qubit HS Assay as follows:
  a. If needed, retrieve RRBS Library samples from −20°C.
  b. Quantify the RRBS Libraries using Qubit Assay, as described at Step 17.
42. Carry out the Fragment Analyzer analysis as follows:
  a. Remove DNF-474 HS NGS Fragment Analysis Kit reagents from storage. Place the Dye, Diluent Marker, and DNA Ladder on ice, and place the remainder at 20°C–25°C.
  b. Turn on the Fragment Analyzer instrument and wait for at least 30 s before turning on the Fragment Analyzer instrument software.
  c. Prior to the assay, perform a water wash by adding 50 mL of deionized water into the Gel 2 container of the Fragment Analyzer instrument.
    i. On the Fragment Analyzer software, select Utilities → solution levels → update the Gel 2 volume to 50 mL. Then select Utilities → Prime → Gel 2 to prime gel 2.
    ii. Select “Add to Queue,” - > Method A: water wash. Hit the green play button to start the method. This runs for ∼20 min.
    iii. Once the wash is complete, check the waste plate for even well volumes. If they are not even, perform a NaOH flush and a capillary array flush as instructed in the Fragment Analyzer instrument manual. In a 250-mL Conical Bottom Centrifuge Bottle (Fisher Scientific, 50-111-7985), mix 4.0 μL of Intercalating Dye and 40 mL of NGS Fragment Separation Gel. Mix well. Store away from light.
  d. Prepare 100 mL of 1x Inlet Buffer from the 5x Buffer. Dispense 1 mL of 1x Inlet Buffer into each well of a 96-deep well plate (Fisher Scientific, 12-566-611). This 1x Inlet Buffer plate can be reused for up to a day.
    **CRITICAL:** Be sure to use the correct plate type (Fisher Scientific, 12-566-611) at Step 42d, as the Fragment Analyzer is incompatible with most alternate types of deep-well plates.
  e. Dispense 200 μL of 0.25x TE Rinse Buffer to each well of a semi-skirted plate (Eppendorf, Cat.# 951020303). This 0.25x TE Rinse Buffer plate can be reused for up to a day.
    **CRITICAL:** Be sure to use the right semi-skirted plate (Eppendorf, Cat.# 951020303) at Step 42e, as the Fragment Analyzer is incompatible with most alternate types of semi-skirted plates.
  f. Prepare 50 mL of 1x Capillary Conditioning Solution by diluting from the 5x Solution to 50 mL of 1x capillary conditioning solution in a 250-mL Conical Bottom Centrifuge Tube.
  g. Prepare the samples in a separate semi-skirted plate, referred to as the Sample plate:
    i. Add 2 μL of HS NGS Fragment DNA Ladder into well position H12 of the Sample plate.
    ii. Add 2 μL of Library sample per well in the Sample plate (except for position H12).
    iii. Add 22 μL of High Sensitivity NGS Diluent Marker per used well (i.e., containing sample or Ladder) and mix.
    iv. Add 24 μL of Blank Solution into any unused wells.
    v. Centrifuge the Sample plate at 200 × *g* to remove air bubbles.
  h. Add plates into the following trays of the Fragment Analyzer:
    i. Tray B: 1x Inlet Buffer plate.
    ii. Tray W: Waste (Fisher Scientific, 12-566-611).
    iii. Tray M: 0.25x TE Rinse Buffer plate.
    iv. Tray 1: Sample plate.
  i. In the side compartment of the instrument, add the conical bottles of Dye-Gel solution and the Capillary Conditioning Solution. Refill the deionized water in the Gel 2 container to 50 mL if needed. Empty the waste container if needed.
  j. In the Fragment Analyzer software, select Utilities → Solution levels and update solution volumes used for Gel 1 (NGS Fragment Separation Gel), Gel 2 (water), Conditioning Solution, and waste.
  k. Prime by selecting Utilities → Prime → select Gel 1, Gel 2, and Conditioning Solution.
  l. Select Tray 1, select “Add to Queue” - > method DNF-474-(33 OR 55) - HS NGS Fragment 1–6000 bp method, and start the separation method. This requires about 71 min.
  m. Once the separation is complete, open the ProSize software, then open the run folder to view the data. On ProSize, select File → open file, then select the file folder icon. Select OS - > Agilent technologies → Data → select run → open run
  n. Fix capillary alignment positions if necessary. Under Analysis, select ‘view capillary positions’. Align each red dot at the top of a peak. Make sure each well on the plate above has an assigned number.
  o. Determine the average fragment size for each sample. Assign a range for smear analysis by clicking on the tool icon on the bottom right corner, then select ‘smear analysis’. Add a range of 200–1000 bp, then select ‘apply to all’. Each sample should display an average fragment size within that range.
  p. Review the electropherogram profile/Fragment Analyzer trace of each sample in the plate. Refer to Figure 6. Problem 3.
    ***Note:*** Typically, Fragment Size = Average Size for the 200–1000 bp Range.

**Figure 6:**
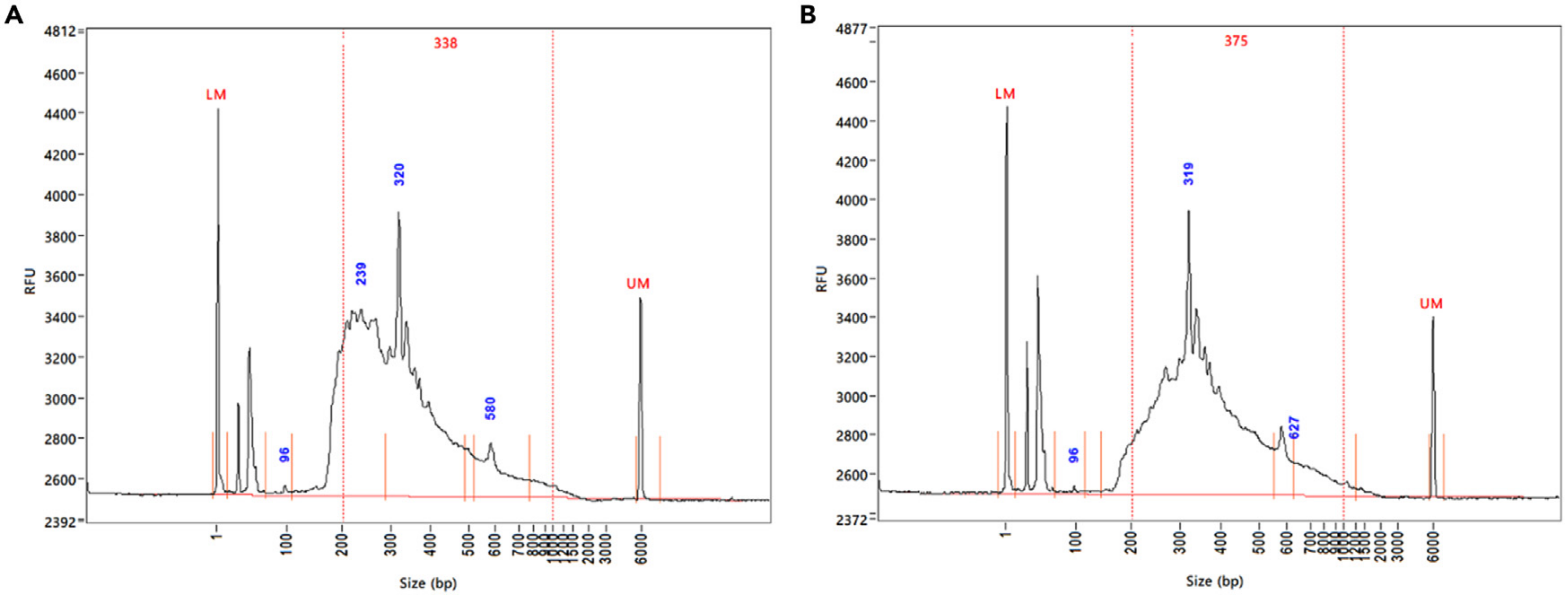
Representative Fragment Analyzer traces of RRBS libraries (A) Fragment distribution of a rat muscle RRBS library that passed QC. There are three characteristic peaks around 240, 320 and 580 bp. (B) Fragment distribution of a rat muscle RRBS library that failed QC. A 1-μL aliquot of the RRBS library was loaded into the gel.
  q. Export the smear analysis excel file and the run PDF file containing the library traces. The smear analysis file will display the average fragment size for each sample.
43. Determine QC status, and calculate the Molarity of Library samples based on Qubit Concentration and Average fragment size from the smear analysis file generated by the Fragment Analyzer.

$$\text{Molarity}\;\left(\text{nM}\right)=\left[\left(\text{Qubit}\;\text{Conc}.\;\text{in}\;\text{ng}/\mu L\right)\;/\;\left(\text{Fragment}\;\text{size}\;\text{in}\;\text{bp}\times 660\right)\right]\times 10^{6}$$

### Library pooling and MiSeq sequencing

**Timing: 1 h 45 min**

RRBS libraries are diluted to a uniform concentration, pooled, and sequenced in a MiSeq Nano flow cell to assess the library quality. Various QC metrics are collected. Low-quality libraries are flagged for library re-generation.

44. Based on the lower molarity samples, choose the final concentration, which all Library samples will be diluted to. Target concentration is 5–10 nM.
45. Calculate the volume of Low-EDTA TE Buffer at pH 8 (10 mM Tris, 0.1 mM EDTA, pH 8.0; Thermo Fisher Scientific, J75793-AE) to add to each Library sample, then dilute each sample accordingly:

VolumeofLow−EDTATEBuffer=[(LibraryMolarity×VolumeofLibrarysample)/DesiredPoolMolarity]−VolumeofLibrarysample

MinimumVolumeofLibrarysample=2μL

***Note:*** MiSeq sequencing requires at least 2 nM of library concentration. Use a minimum of 5 μL Library sample for the dilution.

46. Pool together each of the diluted Library samples, using the same volume for each sample.
47. Verify the Library pool molarity by using the Qubit HS dsDNA assay with a low standard range for standard curve. Run the pool on Qubit in duplicate (Refer to Steps 17, 41).

**Pause point:** The Library pool can be stored at −20°C for several months.

48. For the MiSeq sequencing, follow the steps 39–44 described in our recent STAR protocol publication,^6^ except for the following:
  a. at step 41, for the preparation of PhiX control to include in MiSeq sequencing, make a 10% PhiX spike-in. Please refer to the Illumina ‘MiSeq System Denature and Dilute Libraries Guide, Denature and Dilute PhiX Control’.
  b. Use a custom Read1 sequencing primer (provided with the RRBS library preparation kit) for the MiSeq run:
    i. Prepare custom primer by diluting the sequencing primer (MetSeq Primer 1) provided in the Ovation RRBS Methyl-Seq System (*NuGEN, Catalog #0553*) from 100 μM to 0.5 μM in 600 μL Hybridization Buffer (HT1).
    ii. Load 600 μL of 0.5 μM custom primer into position #12 of the MiSeq reagent cartridge, according to Illumina’s recommendations.
49. When the sequencing is complete, collect the QC metrics (refer to Table 1) and assess the sequencing quality. If needed, adjust sample volumes in the pool based on the results to further balance the sequencing read distribution prior to deep sequencing (problem 4, problem 5, and problem 6).

**Pause point:** The Library pool can be stored at −20°C for several months until Deep sequencing.

### Deep sequencing of RRBS libraries

**Timing: > 36 h**

Sequencing of RRBS libraries is performed on a NovaSeq 6000 platform (Illumina, San Diego, CA, USA), using a paired-end 100 bp run configuration. Pooled libraries are spiked in with 10% PhiX and sequenced to a minimal depth of 40 million paired-end reads per library using a custom 1-index primer as per Illumina guidelines. In addition to the 8-base barcode, the adaptor contains an 8-base unique molecular identifier (UMI) immediately following the library index. The UMIs are used for duplicate read determination (see below). To take advantage of this feature, the libraries are sequenced using 16 cycles for the i7 index read.

50. Follow the steps 45–46 described in our former STAR protocol,^6^ except that the sequencing parameters are as follows:
  a. Read 1 with 100 cycles.
  b. The first index with 16 cycles; 8 cycles for the first sample barcode index (i7 index) and 8 cycles for the UMI).
  c. The second index with 8 cycles for the second sample barcode index (i5 index).
  d. Read 2 with 100 cycles.

### RRBS data processing

**Timing: 1–3 days**

This section summarizes how the sequencing data are processed and subjected to the Molecular Transducers of Physical Activity Consortium (MoTrPAC) RRBS Pipeline, which ends in the collection of the QC metrics.

51. Use bcl2fastq 2.20 software to obtain demultiplexed fastq files from the raw data for each RRBS library sample, as described in the MoTrPAC Genomics, Epigenomics and Transcriptomics (GET) Manual of Procedures (MOP).
52. Use the MoTrPAC RRBS Pipeline to process the RRBS data and collect QC metrics (refer to Table 2). The pipeline is described in the MoTrPAC GET MOP; the pipeline code has been deposited in the GiHub: MoTrPAC/motrpac-rrbs-pipeline and was implemented with the GET snakemake (GitHub: yongchao/motrpac_rrbs). Details of the pipelines mentioned above are provided in our recent preprint.^4^

**Table 2:** The primary metrics used for assessing the quality of the deep sequencing data of RRBS libraries

	Description	Criteria for passing QC
reads_raw	the number of reads in the raw fastq files	>30 million
Predicted gender		Same as sample annotation
Sample identity	Confirm sample identity with other omics data, e.g., RNA-seq	Yes
CpG sites	Number of CpG sites with ≥ 10x coverage	High

Source: The MoTrPAC RRBS MOP.

For reference, see https://github.com/yongchao/motrpac_rrbs and MoTrPAC Study Group 2022^4^ (Supplementary Material, Additional file 1 - Methods).

The same metrics as MiSeq are collected and are expected to pass QC.

### DNA methylation data analysis

**Timing: days to weeks**

This section summarizes the strategy employed to analyze the RRBS sequencing data obtained from various rat tissues and presents typical analysis results.

**Figure 7:**
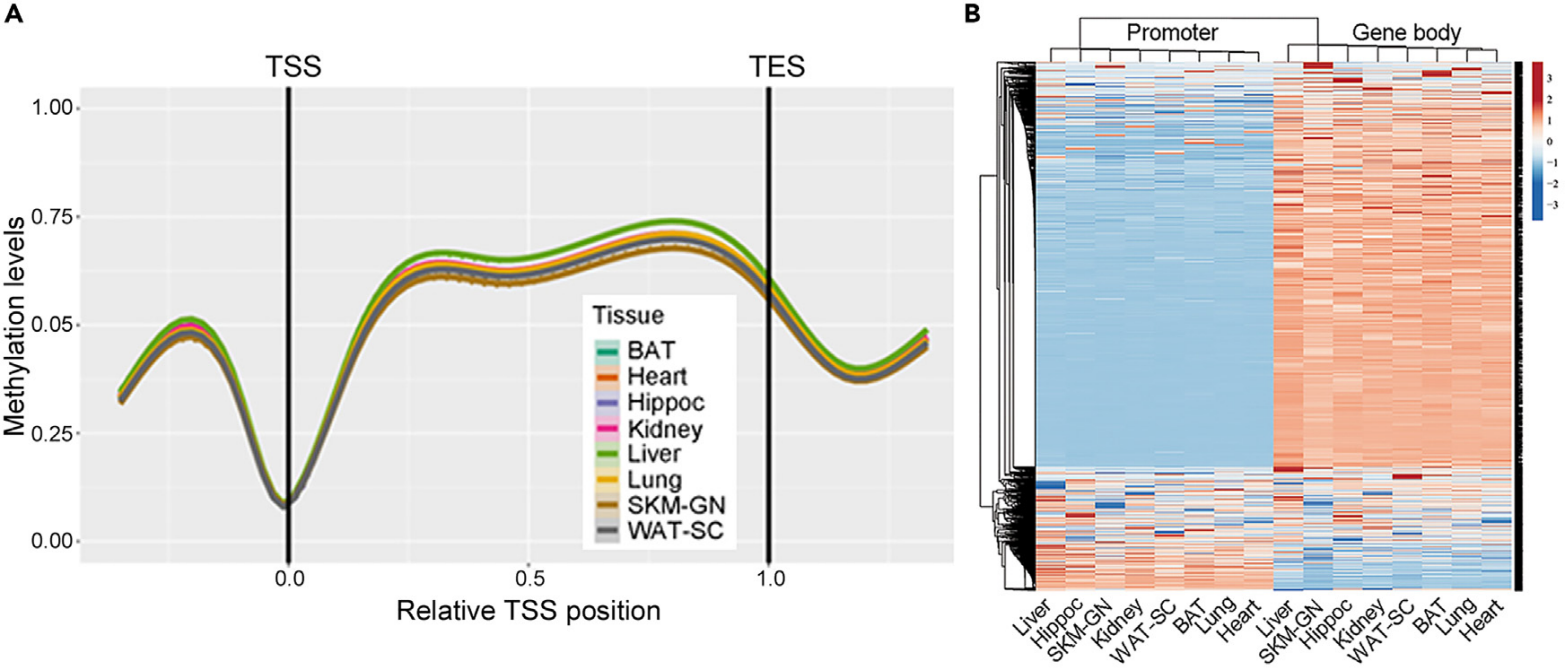
RRBS data analysis (A) Composite plot of the DNA methylation levels averaged across all annotated genes per tissue, generated using RnBeads. Each gene is covered by six equally sized bins and by a 5′ and a 3′ flanking region of similar size. Smoothing is done using cubic splines. Promoters are defined as regions spanning from −1.5 kb to +0.5 kb relative to the transcription start site (TSS). Gene bodies (GBs) are defined as regions extending from TSS to the transcription end site (TES). From Nair et al.^1^, Figure S8. (B) Hierarchical clustering heatmap of promoter and GB methylation levels for all gene features across all eight tissues. Clustering uses complete linkage and Pearson correlation. From Nair et al.^1^, Figure 2G. BAT, brown adipose tissue; Hippoc, hippocampus; SKM-GN, skeletal muscle-gastrocnemius; WAT-SC, white adipose tissue-subcutaneous.

53. Processed RRBS data are filtered, normalized, and analyzed using the R package RnBeads v2.0^6^ in RRBS mode, requiring a minimum coverage of 5 reads, as described in further detail in Nair et al. (2024).^1^ Hierarchical cluster analysis and PCA of average genome-wide DNA methylation levels allow to separate samples by tissue type and sex and visualize the global DNA methylation landscape across 8 rat tissues (Figure 7).

## Expected outcomes

Following DNA extraction, DNA yields are expected to range anywhere between 10 and 400 ng/μL, depending on the tissue type. Similarly, RRBS library concentrations can range anywhere between 1 and 60 ng/μL, depending on the tissue type.

The fragment size distribution of RRBS libraries is expected to show two spikes or peaks around 320 and 580 bp, due to MspI-containing micro-satellite repeats (Figure 6).

As for the expected outcomes for MiSeq and DeepSeq, please refer to their respective QC metrics in Tables 1 and 2. In Table 3, we summarize the NovaSeq outcomes from male rat gastrocnemius samples. All the RRBS data derived from eight rat tissue types are accessible through the MoTrPAC Data Hub (https://motrpac-data.org/data-access) and have been deposited at GEO (GSE242358). Additionally, we were able to characterize the DNA methylation landscapes of these eight rat tissues (Figure 7) and identify differentially methylated regions (DMRs) across tissues.^1^

**Table 3:** List of metrics used for the detailed assessment of the deep sequencing data

QC parameters	Sample 1	Sample 2	Sample 3	Sample 4	Sample 5	Sample 6	Sample 7	Sample 8	Sample 9	Sample 10
Sampled ID	90217016604	90218016604	90222016604	90223016604	90225016604	90227016604	90229016604	90232016604	90237016604	90239016604
reads_raw	30744479	35192545	32546148	29617525	33490305	47137104	35544095	44124133	35476858	29649269
pct_adapter_detected	74.55	73.1	75.55	74.3	74.15	72.6	73.75	74.8	73.25	72.5
pct_trimmed	1.036	1.032	1.097	1.098	1.072	1.085	1.092	0.982	2.67	2.972
pct_no_MSPI	5.11	5.914	4.981	5.732	5.233	5.212	5.107	5.247	6.16	6.777
pct_trimmed_bases	23.303	21.785	23.994	22.954	22.519	21.356	22.171	23.427	22.913	22.252
pct_removed	6.093	6.885	6.023	6.767	6.25	6.241	6.143	6.177	8.666	9.548
reads	28871199	32769544	30585849	27613367	31397284	44195345	33360768	41398425	32402454	26818378
pct_GC	30	30	30.5	30	30	29.5	30	30	30	30
pct_dup_sequence	72.31	73.191	73.057	71.386	73.083	75.632	73.186	75.363	73.122	70.971
pct_chrX	1.179	1.186	1.173	1.181	1.177	1.181	1.184	1.172	1.191	1.187
pct_chrY	0.03341	0.03489	0.03274	0.03404	0.03447	0.03502	0.03505	0.03222	0.03548	0.03471
pct_chrM	0.037	0.038	0.033	0.033	0.034	0.037	0.035	0.034	0.037	0.037
pct_chrAuto	98.198	98.172	98.217	98.188	98.201	98.174	98.175	98.213	98.171	98.166
pct_contig	0.553	0.569	0.544	0.564	0.554	0.572	0.57	0.548	0.566	0.575
pct_Uniq	66.364	66.434	66.572	66.327	66.649	66.964	66.956	66.551	67.063	67.092
pct_Unaligned	13.677	14.043	13.323	13.992	13.881	13.93	13.752	13.811	13.641	13.985
pct_Ambi	19.96	19.524	20.105	19.681	19.47	19.106	19.292	19.638	19.296	18.923
pct_OT	50.014	50.011	50.004	50.015	50.021	50.012	50.016	50.01	50.006	50.03
pct_OB	49.892	49.907	49.905	49.898	49.9	49.912	49.905	49.898	49.913	49.897
pct_CTOT	0.048	0.042	0.046	0.044	0.04	0.038	0.04	0.046	0.041	0.037
pct_CTOB	0.046	0.04	0.045	0.043	0.039	0.038	0.039	0.046	0.04	0.036
pct_umi_dup	9.329	9.867	9.488	9.273	9.202	9.62	9.366	9.403	9.482	9.396
pct_CpG	34.608	35.638	35.151	35.677	35.104	36.085	36.024	33.732	36.164	37.035
pct_CHG	0.7	0.686	0.711	0.796	0.675	0.693	0.741	0.642	0.74	0.721
pct_CHH	0.518	0.515	0.526	0.56	0.498	0.509	0.55	0.473	0.549	0.532
lambda_pct_Uniq	0.24	0.226	0.298	0.299	0.34	0.235	0.323	0.172	0.286	0.324
lambda_pct_umi_dup	10.156	10.678	10.051	10.022	9.936	10.42	10.155	9.982	10.293	10.374
lambda_pct_CpG	0.47	0.458	0.459	0.526	0.434	0.475	0.496	0.442	0.496	0.47
lambda_pct_CHG	0.584	0.564	0.594	0.702	0.55	0.568	0.618	0.543	0.623	0.577
lambda_pct_CHH	0.461	0.445	0.439	0.506	0.42	0.458	0.477	0.454	0.491	0.446

Shown are the QC metrics for RRBS libraries generated from 10 rat skeletal muscle samples.

## Limitations

The automation of genomic DNA extraction and RRBS library preparation clearly has significant benefits over hand-operated procedures, namely: i) protocol standardization, ii) increased accuracy and efficiency, iii) higher throughput, iv) reduction in personnel costs. Nevertheless, our protocol also presents a few limitations.

Inherently, RRBS is a high-resolution, cost-effective technique for genome-wide mapping of DNA methylation, which targets CpG-rich regions like CpG islands, yet it lacks coverage of low-CpG-density genomic regions such as intergenic and distal regulatory regions.^7^ Thus, RRBS identifies differentially methylated regions (DMRs) in higher density CpG regions (≥3 CpG/100 bp), but is not effective for the majority of the genome with lower CpG densities.^8^

From a technical standpoint, the use of an automated workstation can be associated with risks such as instrument failure, especially after prolonged idle times, or power outages. To minimize periods of instrument downtime, the laboratory personnel require adequate training on the liquid handling station.

From a computational point of view, the RRBS data QC pipeline is highly dependent upon the availability of high-performance computers. Without this powerful resource, one cannot process and analyze the sequencing data.

## Troubleshooting

### Problem 1

The extracted DNA has poor quality, i.e., low ratios of 260/230 and 260/280 UV absorbance (related to Step 16).

### Potential solution

This could be linked to insufficient washing and inadequate removal of impurities causing bead clumping. Consider adding additional Wash steps with WBC Buffer (related to Step 13). DNA samples can also be further purified using the Zymo DNA clean and concentrator kit.

### Problem 2

The DNA yield following genomic DNA extraction from tissue is too low (related to Step 17).

### Potential solutions

• If tissue samples are of poor quality, allow beads to separate from contaminants on the magnetic separator for another 10 min (related to Step 11).
• Consider performing a heated elution at 37–55°C to ensure a complete elution (related to Step 14).
• The eluted DNA can also be further concentrated using a Speed Vac concentrator or the Zymo DNA clean and concentrator kit (Zymo Research, D4029; related to Step 14).
• If more sample tissue is available, consider repeating the DNA isolation.

### Problem 3

The Fragment Analyzer profile of the RRBS library QC is inadequate (related to Step 42; refer to Figure 6).

### Potential solution

The only solution is to repeat the RRBS protocol Parts I and II (related to Step 25). If diluted DNA is no longer available, dilute more DNA from the stock DNA. If stock DNA is no longer available, more DNA should be isolated from tissue if possible.

### Problem 4

The bisulfite conversion efficiency in the MiSeq QC metrics (for the unmethylated Lambda DNA) is below 95%, indicating an overall low C-to-U conversion efficiency (refer to Table 1; related to Step 49).

### Potential solution

Repeat the RRBS library preparation (RRBS Parts I and II; related to Step 25).

### Problem 5

The % GC in the MiSeq QC metrics is abnormal (refer to Table 1; related to Step 49).

### Potential solution

Repeat the RRBS library preparation (RRBS Parts I and II; related to Step 25).

### Problem 6

Other MiSeq QC metrics are substandard, e.g., a low % of uniquely mapped reads or a high duplication rate (refer to Table 1; related to Step 49).

### Potential solution

Adjust the library pool with more sample volume or prepare a new library pool as needed, then repeat MiSeq sequencing. If the problem arises again, consider repeating the RRBS library preparation (RRBS Parts I and II).

## Resource availability

### Lead contact

Further information and requests for resources and reagents should be directed to and will be fulfilled by the lead contact, Venugopalan Nair (Venugopalan.Nair@mountsinai.org).

### Technical contact

Questions about the technical specifics of performing the protocol should be directed to and will be answered by the technical contact, Venugopalan Nair (Venugopalan.Nair@mountsinai.org).

### Materials availability

This study did not generate new unique reagents. Feel free to contact the lead contact with any questions.

### Data and code availability

The RRBS dataset generated in the present study has been deposited in GEO: GSE242358. Processed data and analysis results have been deposited in GitHub and Zenodo. All original code has been deposited in Github and Zenodo. Accession numbers, DOIs, and URLs are listed in the key resources table. Any additional information required to reanalyze the data reported in this paper is available from the lead contact upon request.

## References

[1] Nair V.D., Pincas H., Smith G.Z., Ge Y., Amper M., Vasoya M., Chikina M., Sun Y., Raja A., Mao W. (2024). Molecular adaptations in response to exercise training are associated with tissue-specific transcriptomic and epigenomic signatures. Cell Genomics.

[2] Schübeler D. (2015). Function and information content of DNA methylation. Nature.

[3] Gu H., Smith Z.D., Bock C., Boyle P., Gnirke A., Meissner A. (2011). Preparation of reduced representation bisulfite sequencing libraries for genome-scale DNA methylation profiling. Nat. Protoc..

[4] Amar D., Gay N.R., Jean Beltran P.M., Adkins J.N., Almagro Armenteros J.J., Ashley E., Avila-Pacheco J., Bae D., Bararpour N., Burant C. (2024). Temporal dynamics of the multi-omic response to endurance exercise training.. Nature.

[5] Müller F., Scherer M., Assenov Y., Lutsik P., Walter J., Lengauer T., Bock C. (2019). RnBeads 2.0: comprehensive analysis of DNA methylation data. Genome Biol..

[6] Mendelev N., Zamojski M., Amper M.A.S., Cheng W.S., Pincas H., Nair V.D., Zaslavsky E., Sealfon S.C., Ruf-Zamojski F. (2022). Multi-omics profiling of single nuclei from frozen archived postmortem human pituitary tissue. STAR Protoc..

[7] Yong W.-S., Hsu F.-M., Chen P.-Y. (2016). Profiling genome-wide DNA methylation. Epigenet. Chromatin.

[8] Beck D., Ben Maamar M., Skinner M.K. (2022). Genome-wide CpG density and DNA methylation analysis method (MeDIP, RRBS, and WGBS) comparisons. Epigenetics.

